# Exploring confusion with antibiotic identification at the community level in six low-and-middle-income countries across Asia and Africa

**DOI:** 10.1186/s12889-026-27705-6

**Published:** 2026-05-14

**Authors:** Samuel Afari-Asiedu, Annelie A. Monnier, Nga T. T. Do, Kwaku Poku Asante, Wasif Ali Khan, Mohammad Abdul Matin, Khátia Munguambe, Helena Boene, Esperanca Sevene, Sureeporn Punpuing, Malee Sunpuwan, Tuyet P. Anh, Toan K. Tran, Georgina Pujol-Busquets Guillén, F Xavier Gómez-Olivé, Floidy Wafawanaka, Heiman F. L. Wertheim, the ABACUS II consortium, Paul N. Newton, Céline Caillet, Proochista Ariana, Taniya Sharmeen, Olga Cambaco, Chuc T. K. Nguyen, Wipaporn Jarruruengpaisan, Sizzy Ngobeni, H. Rogier van Doorn

**Affiliations:** 1 Kintampo Health Research Centre, Research and Development Division of Ghana Health Service, Kintampo, Ghana; 2 Department of Medical Microbiology and Radboudumc Community for Infectious Diseases, Radboudumc, Nijmegen, 6525 GA The Netherlands; 3 Oxford University Clinical Research Unit, Hanoi, Vietnam; 4 International Centre for Diarrhoeal Disease Research, Dhaka, Bangladesh; 5 Faculty of Medicine, Eduardo Mondlane University, Maputo, Mozambique; 6 Manhiça Health Research Centre, Manhiça, Mozambique; 7 Department of Family Medicine, Hanoi Medical University, Hanoi, Vietnam; 8 Institute for Population and Social Research, Mahidol University, Nakhonpathom, Thailand; 9 MRC/Wits Rural Public Health and Health Transitions Research Unit (Agincourt), School of Public Health, Faculty of Health Sciences, University of the Witwatersrand, Johannesburg, South Africa

**Keywords:** Antimicrobial resistance, Antibiotics, Responsible antibiotic use, Community-setting, Appearance of oral antibiotics, Medicine dispensing, Antibiotic identification

## Abstract

**Background:**

Antimicrobial resistance (AMR) is largely attributable to inappropriate antibiotic use particularly in low-and-middle-income countries (LMICs). Misidentification of antibiotics could contribute to inappropriate use, as people may use the wrong drugs for their conditions. Misidentification and inappropriate use are worsened by self medication and easy access to antibiotics that are obtained from unapproved sources, including over-the-counter medicine sellers. This study explored community perceptions of confusion with antibiotics identification and pathways to improve the identification through physical or visual appearance.

**Methods:**

A qualitative exploratory study was conducted from June 2021 and December 2022. A total of 140 In-depth interviews (IDIs) with medicine suppliers and 76 focus group discussions (FGDs) with community members were conducted in six countries spanning Africa (Ghana, Mozambique, South Africa) and Asia (Bangladesh, Thailand, Vietnam). Data was thematically analysed, and results presented as narratives with excerpts to support the findings.

**Results:**

Identification of antibiotics through physical and visual appearance emerged as a complex issue influenced by a variety of factors including colours, shapes, sizes, packaging, local terminologies, and cultural beliefs. Visual cues serve as a primary means of antibiotic identification across all studied countries. This is especially significant in contexts where language barriers or low literacy hinder identification by written labels. While there are common trends across the studied countries, such as the importance of colour in identification, there are also significant regional and local variations that should be considered.

**Discussion:**

The study highlights visual cues a primary means of antibiotic identification, providing valuable insights for developing targeted health literacy and antibiotic awareness interventions. Ultimately, enhancing the ability of community members to accurately identify antibiotics is a key step in promoting responsible antibiotic use practices and improving global health outcomes.

**Supplementary Information:**

The online version contains supplementary material available at https://doi.org/10.1186/s12889-026-27705-6.

## Background

Antimicrobial resistance (AMR) is a growing global health challenge particularly in low- and-middle-income countries (LMICs) where the burden of infectious diseases is high and access to appropriate healthcare is limited [1–3]. A major driver of AMR is inappropriate antibiotic use, influenced by various contextual factors especially at the community level [4, 5]. Studies on inappropriate antibiotic use have largely focused on gaps in community knowledge about when and how antibiotics should be used [6–8]. While such focus have sought to generate useful interventions to address community antibiotic use, they risk obscuring contextual understanding about the ways in which medicines are *known*, identified, and mobilized outside formal biomedical systems [9, 10].

Understanding how community members identify and relate to antibiotics requires attention to the perceptual and experiential heuristics that shape everyday medicine use practices especially in settings with different languages and limited health literacy. Cognitive heuristics, the mental shortcuts through which people make rapid judgments under uncertainty, works more in contexts where formal pharmaceutical knowledge is inaccessible or absent [11, 12, 13]. In such settings, the physical appearance such as color, shape, and size become primary resources through which medicines including antibiotics are recognized, remembered, and distinguished from other medicines. This draws attention to the semiotics of pharmaceuticals i.e. the ways in which physical attributes function as signs carrying meaning that guides health decision-making beyond their pharmacological identity [14, 15]. Also, the knowledge that communities bring to identification of medicine is not just cognitive but embodied in memory and practical experience [16, 17]. Whilst these frameworks shift the focus from what communities think about antibiotics to how they recognize and make sense of them in their daily lives, it also creates confusion with identification.

Findings from the AntiBiotic ACcess and USe (ABACUS 1) project (www.abacus-project.org) revealed confusion with the identification of antibiotics. The study revealed that communities in Ghana and Mozambique largely rely on physical appearance to identify antibiotic as there are no local words or terminologies for antibiotics [18]. ABACUS is a multi-country project that investigated community-based antibiotic access and consumption practices across communities in Asia (Vietnam, Thailand, and Bangladesh) and Africa (Ghana, Mozambique and South Africa) to identify targets and inform community-based interventions to improve antibiotic access and use. In Ghana and Mozambique, antibiotics were identified with shape (capsule) and colours such as red, yellow and red, black and red [19, 20]. In Ghana, amoxicillin and tetracycline were generally referred to as ‘red and yellow’ which may be confusing as both capsules have similar colours and could be used interchangeably [19]. Antibiotics are often perceived in the community as 'strong' medicine that can treat a wide variety of diseases [6, 9], and a study in central Ghana found that antibiotics were often used to treat non-bacterial infections such as muscle pain [6]. The challenge of misidentification and inappropriate use is exacerbated by self-medication and easy access to antibiotics that are obtained from unapproved sources including over-the-counter medicine sellers (OTCMS) with or without prescription. In Ghana and Mozambique, antibiotics are frequently obtained from over-the-counter medicine sellers and informal sources without prescription. In South Africa, while formal pharmacy systems exist, dispensing without prescription remains common in some communities. In Bangladesh and Vietnam, antibiotics are widely available over the counter in both urban and rural settings. In Thailand, regulatory frameworks are comparatively stronger, though informal access persists particularly in rural areas. These differences in access pathways are likely to shape how communities develop identification strategies and navigate pharmaceutical choices at the community level.

Relying on physical appearance—which includes any visually perceptible attributes identifiable by consumers or healthcare providers—could lead to confusion in identifying oral antibiotics and confusing antibiotic with other medicines due to variety in appearance [1, 19]. This notwithstanding, there is an opportunity to leverage and standardise the physical appearance of antibiotics to promote awareness about appropriate use and AMR (especially non-literates) and suppliers as this remains a relatively unexplored strategy. Improving awareness about appropriate antibiotic use and AMR through effective communication and education is one of the objectives of the WHO Global Action Plan on AMR (GAP-AMR) [21, 22], supported by the annual World Antimicrobial Awareness Week (WAAW). During the yearly WAAW, governments and relevant stakeholders engage in several risk communication activities on inappropriate antibiotic use and AMR [22–24]. It is therefore important to further understand the contextual issues around physical appearance for antibiotics identification in LMIC and explore the potential of leveraging physical appearance to improve awareness among communities [18]. Communities or patients’ abilities to identify antibiotic is important for any future intervention aimed at improving antibiotic use and awareness about AMR.

This study explored community perceptions of confusion with antibiotics identification and pathways to improve the identification through physical or visual appearance also contributing to a broader understanding of how medicines are known and recognized outside formal biomedical systems.

## Methods

### Study setting

This study was conducted in rural and semi-urban communities (hereafter referred to as communities) of six demographic surveillance sites in countries which participated in ABACUS I [1, 25] based on their income status in 2013 using the World Bank classification of LMIC criteria for 2014 [26]. These countries included one low-income country from Africa (Mozambique) and three lower middle-income countries from Africa and Asia (Bangladesh, Vietnam, Ghana), and two upper middle-income countries (South Africa and Thailand). It was important to include all study sites of ABACUS I project to further understand the contextual complexities and cultural norms that could influence antibiotic identification through physical appearance [1, 25]. The study sites have been described in detail in a previous paper [1, 25].

### Design

A qualitative exploratory research was conducted from June-2021 to December-2022 in two phases. First, IDIs and FGDs were conducted among community members and medicine dispensers to understand perception regarding physical appearance of antibiotics. In the second phase, additional FGDs and IDIs were conducted on potential impact of standardising physical appearance of antibiotics to improve identification. The results are presented following the COREQ criteria for reporting qualitative research [27].

### Data collection

The IDIs and FGDs were conducted in two phases in Ghana and Bangladesh. South Africa participated in phase two data collection. Mozambique, Thailand and Vietnam combined phases one and two to reduce multiple contacts with participants due to COVID-19. IDI and FGD interview guides (Appendix 1) were developed based on previous experiences from the researchers, existing literature and published studies from ABACUS consortium [28].

#### IDIs with antibiotic dispensers

A total of 140 IDIs were conducted with medical doctors, nurses, midwives, community health-workers, pharmacists, and village grocers/vendors (Table 1). Antibiotics dispensers were purposively selected to represent the diverse range of dispensers in the respective study sites. It was important to include antibiotic dispensers from public and private facilities as well as approved and unapproved dispensers outlets, because of the existence of plural health systems at the study sites, where both government and private institutions dispense antibiotics. At these study sites, antibiotics could also be obtained from unapproved informal dispensers including OTCMS with or without prescription. These informal dispensers live in the communities with patients and are usually the first point of call when they are unwell. The IDIs therefore sought to understand the perspectives of dispensers on patients’ confusing antibiotics with other medicines and how to improve the identification through physical or visual appearance for improved patience education and awareness about AMR. Dispensers were at least 18 years old and actively dispensing or selling antibiotics, as confirmed during a mapping process. All dispensing outlets, formal or informal, licensed or unlicensed, that were mapped during the ABACUS 1 project was updated. Potential participants/dispensers were contacted and the aim of the study was explained to them. Dispensers who were willing to participate and provided consent were then interviewed.

**Table 1:** Number of IDIs and FGDs conducted across ABACUS II study sites*

Country	Types of IDI Participants	Number of IDIs (*n* = 140)	Number of participants in the IDIs (*n* = 140)	Types of FGD groups	Number of FGDs (*n* = 76)	Total number of participants in FGDs (*n* = 643)
Bangladesh	Medicine seller/dispenser (31) Nurse prescriber at government health centre (1)	32	32	Mixed group (men and women) (1) Men groups (8) Women groups (9)	18	145
Vietnam	Local medicine sellers (15) Pharmacists at community health centre (5)	20	20	Mixed groups (3) Men group (1) Women groups (6)	10	89
Thailand	Pharmacists (8) Nurses (6) Doctor (1) Public health worker (1) Village grocery shop owners (4)	20	20	Mixed group (men and women) (1) Men groups (4) Women groups (5)	10	80
Ghana	Over the counter medicine sellers (14) Community Health Officers (2) Midwives (3) Pharmacist (1) Pharmacy Technician/Assistant (3)	23	23	Men groups (8) Women groups (4)	12	89
Mozambique	Pharmacists from private pharmacy (7) Pharmacists from public health facility (18) Pharmacists from private clinics (2)	27	27	Men groups (7) Women groups (9)	16	152
South Africa	Medical doctors (2) Pharmacists (6) Professional nurses (10)	18	18	Mixed groups (men & women) (7) Women groups (2) 1 men group (1)	10	88

^*^Total number of participants in the study = 719, IDI – In depth interviews, FGD – focus group discussion

#### FGDs with community members

A total of 76 FGDs were conducted among 643 community members aged 18 years and above (Table 1). The sample size and number of IDIs and FGDs that were conducted were guided by the principle of informational saturation determined by country teams. The country study teams determined points of saturation during the IDIs and FGDs when subsequent responses from IDI and FGDs were similar to earlier. Participants were purposively selected with each group consisting of 6—8 participants. The study team recruited participants through community liaison persons who were formally employed by the researchers (field staff) or volunteered to support the recruitment process. The liaisons were engaged to contact potential participant to explain the aim of the study to them and to solicit for their participation. Community members who were willing to participate were further engaged and included in the study. Three types of groups including males, female, and mixed group of men and women were recruited for the FGDs. At the study sites, men have significant influence in household healthcare decision making including when, where, and how women and children access healthcare. Women are the primary caregivers and health seekers and often manage day-to-day health issues for children and family members. It was therefore important to understand the perspectives of men and women regarding confusion with antibiotic identification and how that could be improved. The mixed group discussions also provided valuable insights and further validated issues that emerged from the separate group discussion. Phase one FGDs were conducted among selected community members to explore their opinions on the appearance of oral antibiotics and standardization of appearance to facilitate identification. Phase two FGDs focused on the potential impact of standardizing the physical appearance of antibiotics.

#### Study procedures for IDIs and FGDs

The IDIs and FGDs were held in the local language across the different countries: Ghana (English and Twi), Mozambique (Portuguese and Changana), South-Africa (Xitsonga), Bangladesh (Bengali), Thailand (Central Thai dialect) and Vietnam (Vietnamese). Data collection procedures were standardised across the study site. IDIs were conducted face-to-face mostly at the premises where medicines were dispensed to patients/clients. IDIs lasted for about 45 min and were conducted during hours when patients/clients attendance was very low to avoid interruption. FGDs were conducted at a place of convenience for respondents and lasted for about 60 min. Each FGD session was made up of 6 to 8 participants, who were given numbers or pseudo names to ensure anonymity. IDIs and FGDs were audio recorded and facilitated by experienced qualitative researchers at the study sites. The facilitators were taken through qualitative fieldwork and data collection training by the lead social scientist at each site. The facilitators conducted the IDI and FGDs with note-takers who observed proceedings whilst taking notes from the interviews/discussions. The facilitators used the interview guides with specific questions under the various themes but also asked follow-up questions and probed emerging issues. IDI and FGD guides included the following themes: perceptions of antibiotic appearance, enablers of antibiotic identification, barriers to antibiotic identification, suggestions to improve antibiotic identification, and anticipated consequences of easy identification of antibiotics. During data collection in phase two, a brief video (< 2 m 30 s Identify an antibiotic (youtube.com)) was used to nudge discussions and reflections to make abstract concepts (identification of antibiotics) more tangible and relatable for the study participants. The video and subtitles were translated to match the languages in which IDIs and FGD were conducted. Story telling was used to introduce an agriculture analogy (two seeds can look similar but lead to two different crops; in the same way two medicine that look alike can have different actions in the body). The full script of the video is shown in English in (Appendix 2). Facilitators and interviewers showed hard copy flash cards (screenshots from the video) to further illustrate the concepts introduced in the video. The timing of data collection in each study site considered the then ongoing COVID-19 pandemic at the time and implemented safety measures in line with local health regulations.

### Data management and analysis

All audio recordings of IDI and FGD conducted in local languages at the study sites were transcribed verbatim and then translated into English language by trained researchers where applicable. The transcripts were carefully reviewed and edited where necessary to ensure that they reflect the audio recordings. The qualitative data management and analysis software (NVivo 12) was used for the coding of transcripts. A thematic framework approach consisting of five steps [29–31] was used to analyze the data. First, researchers at the study sites read the transcripts and notes (familiarization) to identify recurrent themes. Second, following the data familiarization, a framework was created in English by two senior researchers coordinating the qualitative study based on the recurrent and a priori themes derived from broader topics that had been discussed during IDIs and FGDs. The framework was reviewed and adopted by the study sites whiles allowing emerging codes to be inductively captured during coding across the sites. Third was indexing where the thematic framework was applied consistently to all data. In the fourth step, data from the transcripts were lifted and placed within the appropriate thematic categories/codes within the framework. At each study site, two researchers independently coded first five transcripts and discrepancies were discussed and resolved in team meetings. One researcher per study site coded the remaining transcripts to ensure systemic data analysis. The use of single coders at each site was a pragmatic decision necessitated by the availability of double coders throughout the period of coding. To mitigate potential variability introduced by this approach, all single-coded transcripts were subjected to periodic review by a second researcher and discussed during consortium meetings. This was followed by an interpretive analysis of collated codes under various themes. The findings were presented as narratives and supported with excerpts or quotes from the respondents. The illustrative quotes shown in this manuscript were translated to English and back translated by the study teams to ensure reliability. Regular consortium meetings were held to ensure the preliminary findings were discussed to ensure rigor of the analytic process.

## Findings

This section is categorized into six sub-sections: (i) Demographic characteristics of participants (ii) perceptions of antibiotic appearance (iii) enablers of antibiotic identification (iv) barriers to antibiotic identification (v) suggestions to improve antibiotic identification and (vi) anticipated consequences of easy identification of antibiotics.

### Demographic characteristics of participants

Of the 643 community members who participated in the FGD, 291 (45%) were males and 352 (55%) were females. Participants were between the ages of 18 to 85 years. The employment status of participants were as follows: Farmers 147 (23%), unemployed 134 (20.7), housewife 106 (16.5%), self-employed 60 (9.3%), students 59 (9.2%), formal/government sector workers 56 (8.7%), traders 49 (7.6%), retired participants 32 (5%). For IDI, out of 140 health workers, 75 (53.6%) were males and 65 (46.4%) were females between the ages of 22 to 75 year.

### Perceptions of antibiotic appearance

Community members primarily perceive antibiotics in three main forms: tablets, capsules (mainly for adults), and syrups/powder (mainly for children). The specific attributes associated with these forms varied between countries. Across study sites, these perceptions reflected structured heuristics that is stable and culturally embedded shortcuts through which communities assign identity and meaning to antibiotics. In Bangladesh, antibiotics were commonly perceived as large, longer looking thick tablets predominantly with white colour.

*"The antibiotic powder, which is white in colour, you know. It is in the bottle, we give this to our children…"* (FGD#3 with women, Bangladesh).

In Thailand and Vietnam, antibiotics were most commonly associated with capsules, but some perceive it as tablets. The study reveals a broader range of colours associated with antibiotics, including "white, white-blue, red–black, and yellow red.

*"It was white-blue capsule that I got” (*FGD#7 with Men, Thailand).

*“ It depends on their understanding and beliefs. When they get treated at different health facilities, the appearance of the drugs is different. They do not necessarily know what the medicine is. They may want antibiotics, but they won’ t know whether antibiotics are dispensed. Some still believe that antibiotics are always in capsules.* (IDI#19 with Dispensers, Thailand)

*“It has different colours. I do not even know what its name is. It has a yellow tip with a red tip, with a white one”* (FGD#2 with women, Vietnam).

In Ghana, a unique local term emerged; "topaye" which refers to two-coloured capsules and how combined colour are also associated with specific conditions or symptoms.

*"There are different types of antibiotics. We have the red and yellow (amoxicillin or tetracycline), which is for stomach pains and wounds"* (FGD#2 with men, Ghana).

*"what I can say is that the community people know when you say "topaye" it means antibiotic; but that is not the end. There are other drugs which are not antibiotics but they are in "tupaye" form which is capsule; it is the capsule they are referring to as "topaye". So, to some level, they think antibiotics are "topaye"; well and good, but going forward, not all antibiotics are "topaye"* (IDI#14 with Dispensers, Ghana)

The emergence of "topaye" illustrates how, in the absence of formal pharmaceutical vocabulary, communities generate their own semiotic systems assigning pharmaceutical identity through physical form rather than chemical or biomedical classification. This is not entirely a knowledge gap but a functional epistemic adaptation to a context of linguistic and informational constraint.

South Africa presents a slightly different perspective, with antibiotics being associated with a wider range of colours including "yellow, green, white or brownish." Interestingly, South African participants also emphasize size and shape of antibiotic pills, describing them as "too big" and "round in shape."

*“Yes, there are antibiotics that are white in color and there are some that are brown and navy, there are some that are brown and maroon and once a patient says I want antibiotics that are brown and maroon we know that they are referring to Amoxil.”* (IDI# 2 with dispenser, South Africa).

*“I can say color [of the medicine/antibiotic] itself has meaning and sometimes dangerous …even the pills if they give you red, white and blue pills you might start by with the red pills thinking that they are too strong. Even if you can read according to the grams you will see that the grams are different and the capacity for red once is high”* (FGD 3, males and females, South Africa).

The South African participant's observation that colour "has meaning" is particularly instructive. It reveals an active semiotic process where communities read physical attributes as signifiers of potency, function, and risk, constructing a lay pharmaceutical logic that works independently of biomedical labelling systems.

### Enablers of antibiotic identification

Several common factors enable antibiotics identification across the different countries studied. These enablers can be categorised into visual cues, naming conventions, and experiential factors. These enablers collectively reveal that community-level antibiotic identification is sustained by a coherent, if informal, system of pharmaceutical knowledge grounded in perceptual heuristics, local semiotics, and embodied experience rather than biomedical literacy.

#### Colours as visual cues

Colour emerged as a primary visual cue for antibiotic identification across all studied countries. In Ghana, the distinctive two-colour capsules are key identifiers.

*"The colour of the drug helps us to identify it and know its function. For example, the red and yellow (this could be amoxicillin or tetracycline) drug helps me to treat my stomach trouble…"* (FGD#2 with Men, Ghana).

*"Based on the colour, we can distinguish them. Also, sometimes when they (clients) come, they say: I am looking for some capsules, one part is green and another part is white; or I am looking for a drug which has big tablets (or capsules) and they are red, sometimes too they say it is white* *"* (IDI# 10, with dispenser, Ghana).

In Thailand, the combination of appearance and colour is particularly important, which further corroborates the importance of visual cues in overcoming language barriers among community members.

*"They (community members) cannot read English, and the medicine is referred to (by the provider) using an English name. Thus, it is easier to remember colour instead of the name"* (FGD#8 with men, Thailand).

Dispensers confirm that the community members’ identify antibiotics by forms and colour, especially when shopping at a pharmacy or medicine store.

*“Yes, the name of the medicine is an English name. It may sound similar to other names of medicines, but those are difficult to remember. Instead, they will remember the colour of the pill or capsule”* (IDI#20 with Dispensers, Thailand)

Also in Thailand, people simply refer to antibiotics by the word in the local dialect "ya kae akseb.”[medicine including antibiotics used to treat inflammations, relief pain, swelling, redness, and infections].

*“They just asked for “ya kae akseb” because they don’t know the difference. They only repeat what others have said.”* (FGD#10 with women, Thailand)

*“Yes, for those who ask for ‘ya kae akseb,’ I usually explain to them the need to differentiate the ailment. Around 10% come and directly ask for ‘ya kae akseb.’ If I determine that the ailment is just inflammation, then I try to dispense NSAID”* (IDI#12 with Dispensers, Thailand)

#### Naming conventions- brand names and shortened version

Brand names and shortened versions of antibiotic names serve as identification enablers in some contexts. In Bangladesh, shortened names like "amoxy" for amoxicillin are commonly used.

*“Most of the people ask medicines by brand name. They (customers) memorize the name and ask for Napa (paracetamol), Seclo (Esomeprazole), Ciprocin (Ciprofloxacin), Azin (Azithromycin) etc.”* (IDI#15 with dispensers, Bangladesh).

*“…Napa (pain killer), Ace (pain killer), Antacid and Zimax (antibiotic) are all similar looking medicines (white tablet) they all look same to me”* (FGD#15 with males, Bangladesh).

Dispensers and some community members in Mozambique mentioned that the most efficient way to identify medicines is through the names and labels in the packaging;

*“…I really use the national medicine formulary and the medicine label, how the bottle is...”*. (IDI#18 with medicine dispensers, Mozambique).

*“The pills come with names…That's why we can see that these pills are cotrimoxazole, paracetamol, ibuprofen, because they have names.”* (FGD#3 with women, Mozambique).

#### Previous experience

Previous experience with using antibiotics is cited as an enabler across several countries. This pattern demonstrates that antibiotic knowledge in these communities is fundamentally embodied, accumulated through repeated practical encounters with medicines rather than through formal instruction. The body's memory of a prior treatment outcome becomes the primary guide for future pharmaceutical decision-making.

In Mozambique, one of the antibiotic dispensers explained.

*"If the person had a cough, then they gave antibiotics, amoxicillin, the next time, the person already knows when I had a cough, the medicine that helped me was amoxicillin, which was an antibiotic, so they go directly to the (private) pharmacy to look for that antibiotic"* (IDI#3 with medicine dispensers, Mozambique).

*“Couple of days back, I consumed ciprofloxacin tablet for diarrhoea, which is familiar to me. It was white and oblong shaped”* (FGD#4 with men, Bangladesh).

***“****At times when you give birth at the hospital, they give you some black capsules to help heal the birth wounds. The moment the medicine finishes, we take the paper (package) of the medicine to go and buy the same medicine. When we get to the drug store, we tell the dispenser that we want the black topaye (capsules). So when they see it, they are able to give it to us”* (FGD#3 with women, Ghana).

### Barriers to antibiotic identification

The study also revealed significant barriers to antibiotic identification including visual similarities, language and literacy barriers, and dispensing practices and packing issues. These barriers are not merely practical inconveniences as they represent structural breakdowns in the semiotic and heuristic systems communities rely on.

#### Visual similarities

The difficulty in distinguishing antibiotics from other common medications based solely on appearance was illustrated in Bangladesh, South Africa, Mozambique and Vietnam.

“*Sometimes customers confuses antibiotics with other medicines…one of my customers consumed antibiotics twice a day thinking it was gastric medicine (antacid) because it lookd similar (both white and oval shaped)* (IDI#18 with dispensers, Bangladesh)

*“…some other antibiotics come in different shape and they (clients) are unable to see weather this is an antibiotic or not an antibiotic. For example metronidazole is round and the shape is similar to the one of panado, so they (clients) won’t recognize that it is not an antibiotic* (IDI#10 with dispensers, South Africa).

*“There are similar drugs, it is very difficult to distinguish like erythromycin and albendazole, they are difficult to distinguish because they have the same colour and shape*” (IDI#1 with dispensers, Mozambique).

“*There was a time like that too. Often, they (clients) confuses antibiotics with antipyretics because they are used to treat coughs and fevers and often have the same shape and colour”* (IDI#1 with dispensers, Vietnam).

#### Language and literacy barriers

The use of English names for antibiotics poses a significant barrier to identification especially in Vietnam, Mozambique and Thailand. The excerpts highlight how language barriers can impede the retention and recall of antibiotic names.

*"I don't even know what the antibiotic name is. I heard it few times, but it was in foreign language (English) I can't even pronounce let alone remember"* (FGD#3 with women, Vietnam).

*“… it comes in English and several languages but the Portuguese part has a little of the information, not many, just a few, I read”* (FGD#5 with women, Mozambique).

*“Yes, the name of the drug is an English name. It may sound similar to other names of drugs, but those are difficult to remember*” (IDI#20 with dispensers, Thailand).

#### Dispensing practice and packaging issues

The removal of medicines from their original packaging creates significant identification challenges. In Mozambique, confusion arises when multiple medicines are dispensed without original packaging, often in a simple ziplock bag. Similarly, in South Africa, the study reveals this dispensing practice can inadvertently create confusion.

*"… we have a green panado and we have other green still for pain and it looks exactly the same. If you put them in ziplock bag, I can't tell you the difference"* (FGD#5 with women, South Africa).

*“When they put it in the little plastic bags [ziplocks] how are we going to read it? He [the dispenser] stays with the bottle, and you did not see what it is written on it. The plastic bag does not say anything… They just write paracetamol, but you can't read anything else”* (FGD#16 with women. Mozambique)

### Suggestions to improve antibiotic identification

#### Quick-Response (QR) codes

Participants across the study sites were generally in favour of the enhanced recognition of antibiotics but expressed concerns that some people with limited skills with digital devices (e.g., older persons) might find it challenging to use. That said, most people will have a family member or neighbour who can help them to read the QR code information.

*“QR codes can help us a lot because nowadays people are using smart phones where they can scan and get the information…”* (FGD#4 with men, South Africa).

*“Well, it is good, but it has limitations; people who cannot access a smart phones or software will not be able to read the QR code* (FGD#4 with women, Thailand).

In Mozambique, while QR code was considered a good initiative, its implementation was questioned due to the limited availability of smart phones among community members.

*“That will be good, but our phones are “mbauenes” (not androids)… it will not work for us“.* (FGD#6 with women, Mozambique)

#### Symbols

The use of an ‘AB’ as an identifiable symbol on antibiotic capsules/tablets was appealing to participants, especially in Ghana and Thailand.

*“The “AB” on the pill would help. If there is no symbol, there will be a problem when the colour is similar to other medicines in the same class, especially capsules. In case of allergy, it is important to differentiate the medicines like ‘AB’”* (IDI#2 with dispensers, Thailand)

*“…it’s not like there should be so many writings on it but maybe “AB” like what was showed in the video. Example M&B 760 tablets [pain killer]; people use the number written on it to identify it. Without it, there will be doubts”* (IDI#2 with dispensers, Ghana).

#### Colours

Generally, community members alluded that using different colour for different antibiotic will improve the identification of antibiotics.

*“… each group of antibiotics should be different from others. For instance, if the Azithromycin should use blue logo, then all pharmaceutical companies should follow, it will help customers and sellers for identification”* (IDI#6 with men, Bangladesh)

*It will be more useful if penicillin is one shade of pink and tetracycline is another shade. That way, we can rule out a medicine if a patient is allergic to it* (FGD#2 with dispensers, Thailand*)*

The preference for standardised symbols and colour coding across all sites is coherent with the broader finding that communities rely on visual heuristics for antibiotic identification.

### Anticipated consequences of easy identification of antibiotics

Both community members and dispensers, alluded that easy identification of antibiotics may increase easy access at the community level;

*“Those who will scan [the QR code] and find that it is antibiotics and works for them, they are more likely to demand it all the time”* (IDI#13 with dispensers, South Africa)

*“When there already that system (AB) and we know how to differentiate one (medicine) from another, it will be difficult for us to keep going to the hospital. We will prefer to go to the (private) pharmacy"* (FGD#4 with women, Mozambique)

However, some respondents, both dispensers and community members, observed that the use of antibiotics would not necessarily increase by using a better identification system but rather make people more cautious.

*“It will reduce the use of antibiotics as we have the information about it because we will know all side effects...”* (FGD#2 with male and women mixed group, South Africa)

*“…people will easily identify that these are antibiotics, and what it does. People will be careful when taking antibiotics because they will know that this is how this medicine functions because of the signs on them, and so they cannot just go and take it like that”* (IDI#6 with OTCMS, Ghana).

The divergence in opinion regarding anticipated consequences reflects a tension between two competing reasoning; one in which improved identification empowers autonomous access, and another in which it promotes informed restraint. This tension underscores that improved identification systems cannot be introduced in isolation but must be accompanied by stewardship messaging that shapes how the new semiotic resources are interpreted and acted upon.

## Discussion

This study explored improving identification of antibiotics through their physical appearance. Central to this study is the notion of confusion with antibiotic identification, a concept that encompasses not simply an error or ignorance but a structurally produced outcome of communities way of identifying medicines including antibiotics. Antibiotics are primarily identified by colour, size, and shape which is currently confusing but could be leveraged to promote awareness about appropriate use and AMR at the community level especially among non-literates in LMIC [18]. It’s important to note that, these identification practices represent perceptual and experiential heuristics thus mental shortcuts community members develop in response to navigating healthcare based on information available to them. Accordingly, our study revealed that easy antibiotic identification is desired but shaped by cultural context, with colour and symbols preferred over digital tools. In LMIC, leveraging context-specific ways of identifying antibiotics for improved awareness creation is important as self medication is common accentuated by easy access to antibiotics from unapproved providers, and weak implementation of pharmaceutical regulations.

Our findings showed significant variations in antibiotic perceptions across different countries. The use of local terminologies like "topaye" in Ghana for two-coloured capsules and "ya kae akseb" in Thailand demonstrate how cultural factors can influence medication perceptions and identification which could be confusing [19, 32, 33]. This findings resonate with anthropological studies on pharmaceuticals, which emphasize the importance of understanding local contexts and terminologies in medication use [34, 35]. Though these terminologies may be confusing, they are not merely linguistic conveniences but they function as semiotic resources through which communities assign meaning to medicines based on their physical characteristics, in the absence of formal pharmaceutical vocabulary [35–37]. Across the two continents, a broad pattern emerges. In African settings particularly Ghana and Mozambique identification relied more heavily on colour-based heuristics and locally generated terminologies. In Asia particularly Thailand and Bangladesh, naming conventions, brand familiarity, and shortened antibiotic names played a stronger role, suggesting relatively greater integration with formal pharmaceutical names. Understanding the local context of medication access and use is important in developing context specific strategies to improve on awareness and appropriate us as in the case of antibiotics.

Colour codes serve as primary means of antibiotic identification across all studied countries which could be confusing. This is especially significant in contexts where language barriers or limited literacy may hinder identification by written labels [18, 19]. In Thailand, the combination of appearance and colour was crucial, especially for those who cannot read English labels, which is a potential risk of misidentification as different medications share similar appearances. In Thailand and Bangladesh, shortened names like "amoxy" for amoxicillin are commonly used, while in Bangladesh, brand names given by manufacturers are frequently used for identification. In Mozambique, names and labels on packaging are considered the most efficient way to recognize medicines. These varied identification practices reflect embodied knowledge accumulated through repeated practical encounters with medicines rather than through formal instructions and underscore why standardised visual systems must be designed with local perceptual understanding in mind [16, 38, 39].

One major obstacle to antibiotic identification is the visual similarity between antibiotics and other medicines [19, 20]. In Bangladesh, it is difficult to differentiate between antibiotics and common pain relievers due to similar appearance. Language is also highlighted as Vietnamese and Mozambicans expressed their struggle with pronouncing and understanding antibiotic names in English language. These results underscore the complex interplay of visual, linguistic, and practical factors that can impede accurate antibiotic identification [19, 20]. From a pharmaceutical semiotics perspective, this confusion arises because the physical signs communities rely on to distinguish medicines are insufficiently differentiated creating conditions where misidentification becomes a structurally predictable outcome rather than an individual error. It is also important, however, to resist framing these barriers purely as failures of community knowledge. The same visual and experiential systems that produce misidentification also sustain functional pharmaceutical navigation in resource-constrained settings. Misidentification is therefore better understood as an intelligible outcome of a coherent classificatory system operating at the community level not as evidence of ignorance [15]

While QR codes was generally favoured for enhancing antibiotic recognition, concerns were raised about accessibility particularly older individuals and those with limited access to digital devices. In Bangladesh, where QR codes are more familiar due to use in different small and medium enterprises, the reception was more positive. The study highlights the potential of QR codes while acknowledging the need to address digital literacy and access issues [40]. The results on QR codes are timely as there is an increasing rate of internet use and smartphones in many LMICs especially in Africa [41]. Also, considering the integration of new technologies to improve the healthcare system & performance in recent times, there are great opportunities to incorporate technological tools such as QR codes into healthcare education to efficiently and effectively share information about antibiotics and AMR [40, 42].

The use of symbols 'AB' abbreviation for antibiotics was widely appealing across countries, especially in Ghana and Thailand. Such symbols could aid in quick identification among consumers and healthcare providers and help reduce medication errors. These results highlight the potential of visual cues in enhancing medication safety and suggest that a combination of approaches might be most effective in improving antibiotic identification across diverse populations and healthcare settings [20, 43].

Our study results revealed a paradoxical tension at the heart of improved antibiotic identification. While improved recognition through symbols, colour code standardization, or digital tools such as QR codes is broadly desired by communities and dispensers, there was widespread concern that easier identification could increase demand and facilitate self-medication, as individuals might readily seek out antibiotics they have found effective in the past. Conversely, different viewpoints suggested that improved identification systems for antibiotics could instead promote more cautious and informed use. These contrasting viewpoints is not just a practical tension. It represents one of the most significant contributions of the study, as it fundamentally challenges the assumption that better knowledge or clearer identification will necessarily lead to more appropriate use [19].

This paradox cannot be adequately understood without situating it within the political economy of pharmaceutical access in LMICs. In contexts where antibiotics could be easily obtained from unapproved sources, weakly regulated, and frequently dispensed without prescription, improved identification will not operate in a neutral environment. Rather, it enters a landscape already structured by commercial interests, regulatory gaps, and community-level demand dynamics that shape how new pharmaceutical knowledge is mobilised [44, 45]. Enhanced recognisability, in such a context, may simultaneously serve stewardship goals and undermine them, empowering communities to make more informed choices while also enabling more targeted self-medication. This two possibility suggests that identification interventions cannot be designed in isolation from broader regulatory and stewardship frameworks. Improving how antibiotics are known must be accompanied by deliberate attention to how that knowledge will be acted upon within existing structures of pharmaceutical access and supply.

### Implications for policy

Understanding how people identify antibiotics, local perceptions and beliefs could help in designing effective context-specific educational and stewardship interventions given the global concern about antibiotic resistance and the need for improved appropriate use [46]. The findings of this study suggest that communities have functional informal systems of pharmaceutical knowledge grounded in perceptual heuristics, local semiotics, and embodied experience. Effective policy interventions should therefore engage with how communities already know antibiotics, rather than assuming a knowledge gap that requires filling with biomedical information. The paradox identified in this study that improved identification may simultaneously promote appropriate use and facilitate self-medication has direct policy implications. Standardised visual systems for antibiotics, however well designed, will not achieve stewardship goals unless introduced alongside regulatory strengthening of over-the-counter antibiotic access, community education on stewardship principles, and engagement with informal medicine suppliers. Policymakers should therefore treat identification improvement as a necessary but insufficient condition for appropriate antibiotic use, embedding it within a comprehensive stewardship strategy that accounts for the political economy of pharmaceutical access in LMIC settings. Our results suggest the need to consider standardised visual systems for antibiotics that are consistent across different manufacturers. We previously drafted recommendations for future labelling campaigns based on recent examples in India and Bangladesh [47]. Future research by relevant stakeholders should focus on testing the effectiveness of standardised visual cues for antibiotic identification across different cultural contexts. Additionally, exploring the impact of improved packaging and labelling on antibiotic recognition and proper use could provide valuable insights for policy development. Ultimately, enhancing the ability of community members to accurately identify antibiotics is a key step in promoting antibiotic stewardship and improving global health outcomes.

### Strengths and limitations

Using the physical appearance of antibiotics remains a relatively unexplored strategy to raise awareness about responsible antibiotic use among both users and suppliers. This study is the first to explore this aspect within communities across six different countries.

Results from this study may not be generalizable beyond the various study settings, as representative sampling was not used. However, the results provide contextual evidence that may inform interventions or further studies on the use of visual cues to improve antibiotic identification in LMIC. The use of agricultural analogy and video during data collection may have introduced generic, simplified features that might not be relatable to local communities. Also, the framing of antibiotics in terms of risks and the desirability of improved identification in the video may have primed participants toward more receptive responses to proposed interventions such as symbols and QR codes than might otherwise have been expressed. This notwithstanding, the use of the video allowed researchers to approach the topic of medicine identification without introducing many complex biomedical terms. The presence of the research team to guide and moderate discussion sessions ensured that key topics and messages were effectively conveyed to participants.

Again, while some sites collected information on participants' education level and age, this was not consistent across all six countries. As a result, stratification of findings by these sociodemographic factors was not possible without introducing analytical inconsistency across sites. Education level and age may significantly influence individuals' ability to identify antibiotics and interpret visual cues. Future multi-country studies of this nature should harmonize sociodemographic data collection across all sites to enable meaningful stratification of findings.

Its is important to note that findings from this study are based on participant perceptions and self-reported practices and not directly observed antibiotic use behaviors. Consequently, causal linkages between misidentification and inappropriate antibiotic use cannot be established from this data and should be interpreted as plausible hypotheses rather than empirically confirmed conclusions. Future research employing observational or mixed methods designs would be needed to establish the behavioral consequences of identification challenges.

## Conclusion

Identification of antibiotics through physical and visual appearance is a complex issue influenced by a variety of factors including colour, shape, size, packaging, local terminology, and cultural beliefs. While there are common ways of identification across the studied countries, there are significant regional and local variations that must be considered. The study highlights visual similarity between antibiotics and other medications, use of English names for antibiotics, and dispensing antibiotic out of the original packaging as major obstacle to antibiotic identification. Enablers of identification included the use of QR codes, symbols, and different colours for different antibiotic. These key enablers and barriers could improve accurate antibiotic identification, providing valuable insights for developing targeted health literacy and antibiotic awareness interventions. Importantly, the study revealed that community antibiotic practices may not be entirely due to knowledge gaps. The confusion or misidentification identified in this study may more accurately represent a situation that is a system of pharmaceutical classification grounded in visual and experiential knowledge. Interventions that fail to engage with this existing epistemic system risk being ineffective, however well designed technically. By leveraging the enablers identified in this study, significant progress could be made in improving antibiotic identification, appropriate antibiotic use and awareness creation about AMR. Although not originally framed within these theoretical traditions, the findings resonate with and contribute to scholarship on cognitive heuristics, pharmaceutical semiotics, and embodied knowledge offering empirical grounding for understanding how medicines are known outside formal biomedical systems.

## Supplementary Information

Supplementary Material 1.

## Data Availability

Data are available from the authors upon reasonable request.

## Abbreviations

AMR: Antimicrobial Resistance
GAP-AMR: Global Action Plan on AMR
FGD: Focus Group Discussions
IDI: In-Depth Interviews
LMIC: Low- and Middle-Income Countries
OTCMS: Over the Counter Medicine Sellers
QR: Quick Response
WAAW: World Antimicrobial Awareness Week

## References

[1] Do NT, et al. Community-based antibiotic access and use in six low-income and middle-income countries: a mixed-method approach. Lancet Glob Health. 2021;9(5):e610–9. 10.1016/S2214-109X(21)00024-3 PMC8050200 33713630

[2] UN. Life-threatening infections on the rise due to drug-resistant bacteria, new WHO report reveals 2022 [2024]; Available from: https://news.un.org/en/story/2022/12/1131527. Cited 2024 15 October

[3] WHO. Global antimicrobial resistance and use surveillance system (GLASS) report: 2021. 2021; Available from: https://iris.who.int/handle/10665/341666.

[4] Byrne MK, et al. The drivers of antibiotic use and misuse: the development and investigation of a theory driven community measure. BMC Public Health. 2019;19:1–11. 10.1186/s12889-019-7796-8 PMC6822443 31666056

[5] Gualano MR, et al. General population’s knowledge and attitudes about antibiotics: a systematic review and meta-analysis. Pharmacoepidemiol Drug Saf. 2015;24(1):2–10. 10.1002/pds.3716 25251203

[6] Wall S. Prevention of antibiotic resistance–an epidemiological scoping review to identify research categories and knowledge gaps. Glob Health Action. 2019;12(sup1):1756191. 10.1080/16549716.2020.1756191 PMC7782542 32475304

[7] Ndaki PM, et al. Drivers of inappropriate use of antibiotics among community members in low-and middle-income countries: a systematic review of qualitative studies. BMC Public Health. 2025;25(1):705. 10.1186/s12889-025-21553-6 PMC11841188 39979890

[8] Afari-Asiedu S, et al. Interventions to improve dispensing of antibiotics at the community level in low and middle income countries: a systematic review. J Glob Antimicrob Resist. 2022;29:259–74. 10.1016/j.jgar.2022.03.009 35342021

[9] Melaku T, Assegid L. Prescription in peril: the sociology of antibiotics and antimicrobial resistance in low resource settings. Discov Soc Sci Health. 2025;5(1):71.

[10] Nayiga, S., Antibiotics in Society: a multi-sited ethnography in rural and urban Uganda. 2022, London School of Hygiene & Tropical Medicine.

[11] Whelehan DF, Conlon KC, Ridgway PF. Medicine and heuristics: cognitive biases and medical decision-making. Irish J Med Sci (1971-). 2020;189(4):1477–84. 10.1007/s11845-020-02235-1 32409947

[12] Riva S, Monti M, Antonietti A. Simple heuristics in over-the-counter drug choices: a new hint for medical education and practice. Adv Med Edu Pract. 2011:59–70. https://www.tandfonline.com/doi/pdf/10.2147/AMEP.S13004. 10.2147/AMEP.S13004 PMC3661247 23745077

[13] Gunasekera YD, et al. Misconceptions of antibiotics as a potential explanation for their misuse. A survey of the general public in a rural and urban community in Sri Lanka. Antibiotics. 2022;11(4):454. 10.3390/antibiotics11040454 PMC9024968 35453206

[14] Glinert LH. Communicative and discursive perspectives on the medication experience. Pharmacy (Basel). 2021;9(1):42. 10.3390/pharmacy9010042 PMC8006053 33671135

[15] Tredinnick-Rowe J, Stanley DE. Semiotics in health and medicine. Bloomsbury Semiot Semiotics Nat Tech Sci. 2023;2:119.

[16] Marchand TH. Making knowledge: explorations of the indissoluble relation between minds, bodies, and environment. J R Anthropol Inst. 2010;16:S1–21.

[17] Akande-Sholabi W, Oyesiji E. Antimicrobial stewardship: knowledge, perceptions, and factors associated with antibiotics misuse among consumer’s visiting the community pharmacies in a Nigeria Southwestern State. J Pharm Policy Pract. 2023;16(1):120. 10.1186/s40545-023-00629-x PMC10566051 37821920

[18] Monnier AA, et al. Is this pill an antibiotic or a painkiller? Improving the identification of oral antibiotics for better use. Lancet Glob Health. 2023;11(8):e1308–13. 10.1016/S2214-109X(23)00258-9 37474237

[19] Afari-Asiedu S, et al. Every medicine is medicine; exploring inappropriate antibiotic use at the community level in rural Ghana. BMC Public Health. 2020;20:1–10. 10.1186/s12889-020-09204-4 PMC7359511 32664902

[20] Cambaco O, et al. Community knowledge and practices regarding antibiotic use in rural Mozambique: where is the starting point for prevention of antibiotic resistance? BMC Public Health. 2020;20:1–15. 10.1186/s12889-020-09243-x PMC7389384 32727445

[21] Mendelson M, Matsoso MP. The World Health Organization global action plan for antimicrobial resistance. S Afr Med J. 2015;105(5):325–325. 10.7196/samj.9644 26242647

[22] WHO. *Global Action Plan on Antimicrobial Resistance*. 2015; Available from: https://www.amcra.be/swfiles/files/WHO%20actieplan_90.pdf. Cited 2021 16 August

[23] Choy CY, Hsu LY. World antibiotic awareness week. Ann Acad Med Singap. 2017;46:413–4. 29288258

[24] WHO. World Antimicrobial Awareness Week. 2015; Available from: https://www.who.int/campaigns/world-antimicrobial-awareness-week.

[25] Wertheim HF, et al. Community-level antibiotic access and use (ABACUS) in low-and middle-income countries: Finding targets for social interventions to improve appropriate antimicrobial use–an observational multi-centre study. Wellcome Open Res. 2017;2. https://www.ncbi.nlm.nih.gov/pmc/articles/PMC5897850/. 10.12688/wellcomeopenres.11985.1 PMC5897850 29707652

[26] Neil Fantom, U.S. The World Bank’s Classification of Countries by Income. 2016; Available from: https://documents1.worldbank.org/curated/en/408581467988942234/pdf/WPS7528.pdf.

[27] Tong A, Sainsbury P, Craig J. Consolidated criteria for reporting qualitative research (COREQ): a 32-item checklist for interviews and focus groups. Int J Qual Health Care. 2007;19(6):349–57. 10.1093/intqhc/mzm042 17872937

[28] ABACUS, P. Scientific publications. Available from: https://abacus-project.org/scientific-publications/.

[29] Braun V, Clarke V. Successful qualitative research: A practical guide for beginners. 1st edition. SAGE Publications. 2013.

[30] Clarke V, Braun V. Thematic analysis. J Posit Psychol. 2017;12(3):297–8.

[31] Teo T. Encyclopedia of critical psychology, vol. 1. New York: Springer; 2014.

[32] Sunpuwan M, et al. Understanding antibiotic use in the community setting in Thailand: does communication matter? PLoS ONE. 2024;19(4):e0298972. 10.1371/journal.pone.0298972 PMC10986969 38564533

[33] Wanida C, Kornkaew C. The meaning of Ya-Kae-Akseb in Lay’s perspective: a case study of Ban Nakhok, Moo 3, Srisongrak Subdistrict, Muang District, Loei Province. Isan J Pharm Sci. 2014;9:93–8.

[34] Petryna A, Kleinman A, Lakoff A. Global pharmaceuticals: ethics, markets, practices. Duke University Press; 2006.

[35] Closser S, et al. The anthropology of health systems: a history and review. Soc Sci Med. 2022;300:114314. 10.1016/j.socscimed.2021.114314 34400012

[36] Whyte SR, Van der Geest S, Hardon A. *Social lives of medicines*. Cambridge University Press; 2003 (1st edition) . : https://www.cambridge.org/gb/universitypress/subjects/anthropology/social-and-cultural-anthropology/social-lives-medicines.

[37] Whyte SR, Van der Geest S. Medicines in context: an introduction. In: The context of medicines in developing countries: studies in pharmaceutical anthropology. Springer; 1988. p. 3–11.

[38] Lindenbaum S, Lock MM. Knowledge, power, and practice: the anthropology of medicine and everyday life. Univ of California Press; 1993.

[39] Cruess RL, Cruess SR, Steinert Y. Medicine as a community of practice: implications for medical education. Acad Med. 2018;93(2):185–91. 10.1097/ACM.0000000000001826 28746073

[40] Karia CT, Hughes A, Carr S. Uses of quick response codes in healthcare education: a scoping review. BMC Med Educ. 2019;19:1–14. 10.1186/s12909-019-1876-4 PMC6896690 31810464

[41] Wang D, et al. Access to digital media and devices among adolescents in sub-Saharan Africa: a multicountry, school-based survey. Matern Child Nutr. 2025;21:e13462. 10.1111/mcn.13462 PMC12208891 37014184

[42] Darwish T, et al. Integration of advanced health technology within the healthcare system to fight the global pandemic: Current challenges and future opportunities. Innov Clin Neurosci. 2021;18(1–3):31. PMC8195559 34150361

[43] Monnier AA, Matin C.S., Kamath A, S, Sharmeen T, et al. What can be learned from Medicine Labelling Initiatives in Asia, with a focus on Antibiotics? J Community Med Public Health. 8;43220:4. 10.29011/2577-2228.100432.

[44] Afari-Asiedu S, et al. To sell or not to sell; the differences between regulatory and community demands regarding access to antibiotics in rural Ghana. J Pharm Policy Pract. 2018;11(1):30. 10.1186/s40545-018-0158-6 PMC6293650 30564369

[45] Afari-Asiedu S, et al. Determinants of inappropriate antibiotics use in rural central Ghana using a mixed methods approach. Front Public Health. 2020;8:90. 10.3389/fpubh.2020.00090 PMC7105730 32266200

[46] WHO. Antibiotic resistance: multi-country public awareness survey. 2015; Available from: https://iris.who.int/handle/10665/194460. 10.3390/antibiotics14060599 PMC12189553 40558189

[47] Travasso C. *India draws a red line under antibiotic misuse*. BMJ. 2016;352:i1202. 10.1136/bmj.i1202. 26926703

