# Supplementary material for: Exploring confusion with antibiotic identification at the community level in six low-and-middle-income countries across Asia and Africa
Source: BMC Public Health. 2026 May 14;26:2710. doi: 10.1186/s12889-026-27705-6 (PMC13625308; doi:10.1186/s12889-026-27705-6)
Supplement: Supplementary file 1 [file 12889_2026_27705_MOESM1_ESM.docx]

**TITLE:** Exploring confusion with antibiotic identification at the community level in six low-and-middle-income countries across Asia and Africa

**Appendix 1:**

Topic guide for In-Depth Interviews (IDIs)

1. What kind of illnesses do your customers come to you wanting help with? What kinds of medicine/antibiotics do you usually dispense to help with these illnesses?
2. If the illness/condition is really severe, what medicine/antibiotic do customers request?
3. Do they come to you with a symptom, asking you for help? How do you help? Would you like to elaborate?
4. Do customers come to you seeking particular medicine/antibiotics? Or particular formulation that looks a particular way? Please provide some examples.
5. When they come to you without symptoms, but with requests for a particular medicine/antibiotic, how would they describe that medicine/antibiotic? Please give examples.
6. Do you have difficulty distinguishing the different medicine/antibiotics you sell? Please provide examples.
7. How do you tell the difference? How do you identify the different medicine/antibiotics? (Probe- is it by shape, is it by formulation, by colour, by size, appearance of the packaging, writing in the packaging, colour coding of the packaging etc.)
8. What If there was more uniformity in a particular kind of medicine/antibiotic (if appearance were to be harmonised)? What is your opinion on that?
9. Would it help? How? What sort of uniformity in appearance would you suggest? Is the colour, the shape, size, or formulation?
10. Would it help if there was writing on the tablet or on the blister pack? What are your thoughts on that?
11. Now, what about the writing in the packet? Does it help in recognizing and dispensing medicine/antibiotic? Can you provide an example?
12. Would it help if there was a code in the package or on the pill itself that you could scan with your phone or a devise, and it would tell you that information about that medicine/antibiotic?
13. Are there alternative ways you think that could help in appropriate use of medicine/antibiotics especially antibiotics?
14. Is there anything else you want to share/add anything to our conversation? Is there anything else you want to tell me or add anything to what you have already said?

Topic Guide for Focus Group Discussions (FGDs):

1. How do recognise what medicine/antibiotics are for what illnesses?
2. How do you decide where to get your medicine/antibiotic [pharmacist or doctor or the healer]?
3. How do you know you have the right medicine/antibiotic?
4. What about colour of the medicine/antibiotic/antibiotic? what is colour telling you? Is there any particular preference for colour? Why?
5. What about the shape of the medicine/antibiotic? How do you feel about a particular shape? Why?
6. What shape tells you? Which shapes helped?
7. What about the formulation (tablet & capsules) of the medicine/antibiotic? How do you feel about a particular formulation? Why?
8. What sort of medicine/antibiotic you use when you/family experience severe illness?
9. Now, what about the writing on the packet? Does it help in recognizing medicine/antibiotic? Can you provide an example?
10. What about the reading the information guide that comes with the medicine/antibiotic packet? Does it help in recognizing medicine/antibiotic? Can you provide an example?
11. Is there anything else you want to share/add anything to our conversation? Is there anything else you want to tell me or add anything to what you have already said?

**Appendix 2: Script of the video used for data collection.**

- Meet Anna. She’s a good girl and likes to help her mother.
- She likes helping in the garden best and she wants to plant some vegetables.
- Anna takes some seeds and plants them carefully in the garden.
- She waters her seeds every day and soon they grow.
- But what is this? Her seed isn’t a vegetable seed, it’s a flower! The seeds looked the same, but one grew a flower and the other grew a vegetable
- One day, Anna feels ill. She has a fever and a cough. It’s a flu and her mother is worried about her.
- Her mother goes to the shop to get some medicine.
- Mothers want to give their children the best medicine when they are sick. Many mothers think antibiotics are the best medicine.
- We know that each sickness needs a different medicine. Sometimes the best medicine is antibiotics, but most of the time it isn’t.
- Anna needs time to get better. Antibiotics won’t help her get better any faster because they only work for some sicknesses.
- In fact, antibiotics can cause side effects like diarrhoea or a rash, which are not fun.
- What her mother can do is try to make Anna comfortable and keep her body strong with healthy food and enough to drink.
- Just like seeds there are many kinds of medicine and many look the same.
- It’s hard to tell what’s on the inside.
- We might be able to help you with that. A special symbol right on the medicine can tell you at once whether it is an antibiotic or not.
- A symbol can mean less confusion, fewer medicine mix ups, and fewer side effects we don’t want. It will be easier to use antibiotics the right way. It is not good to use antibiotics if you do not need them.
- In the future, a QR code on your medicine or its packaging could help you find information about how and when to use antibiotics.
- Growing a flower in the vegetable garden is not such a big problem. Using antibiotics when we don’t need them is. We all want Anna to get better and stay healthy. Giving her the right medicine when she needs it will help her grow big and strong!

**Appendix 3:**

Institutional Review Board and ethics approvals obtained for this study:

| **Institute** | **Name of committee** | **Reference** | **Date of Approval** |
| --- | --- | --- | --- |
| University of Oxford, Oxford, United Kingdom | Oxford Tropical Research Ethics Committee | 566-21 | 17th of November 2021 |
| International Centre for Diarrhoeal Disease Research, Dhaka, Bangladesh | Research Review Committee (RRC) | PR-21088 | 16th of August 2021 |
| Kintampo Health Research Centre, Research and Development Division of Ghana Health Service, Kintampo, Ghana | KHRC Scientific Review Committee | KHRC/ADM/2021-9 | 13^th^ of September 2021 |
| Institute for Population and Social Research, Mahidol University, Nakhon Pathom, Thailand | Institutional Review Board, Institute for Population and Social Research, Mahidol University (IPSR-IRB) | COA. No. 2021/09-173 | 30th of September 2021 |
| Manhiça Health Research Centre, Manhiça, Mozambique | CISM Institutional Health Bioethics Committee | CIBS-CISM/104/2021 | 22th of December 2021 |
| Hanoi Medical University, Hanoi, Vietnam | Hanoi Medical University Institutional Review Board (HMU IRB) | HMUIRB570 | 5^th^ November 2021 |
| Wits Rural Public Health and Health Transitions Research Unit (Agincourt) | University of The Witwatersrand Human Research Ethics Committee (Medical) | M210965 | 5^th^ of September 2022 |
